# Qualitative research influencing guideline and policy: An exemplar of the development of a national school asthma guideline in Malaysia

**DOI:** 10.7189/jogh.14.03027

**Published:** 2024-05-17

**Authors:** Siti Nurkamilla Ramdzan, Ee Ming Khoo, Steve Cunningham, Norita Hussein, Rizawati Ramli, Siti Aishah Senawi, Nursyuhada Sukri, Jayakayatri Jeevajothi Nathan, Asiah Kassim, Azainorsuzila Mohd Ahad, Hilary Pinnock, RESPIRE collaborators

**Affiliations:** 1 Department of Primary Care Medicine, Faculty of Medicine, Universiti Malaya, Kuala Lumpur, Malaysia; 2 NIHR Global Health Research Unit of Respiratory Health (RESPIRE), Usher Institute, The University of Edinburgh, UK; 3 Sektor Pengurusan Pembelajaran, Bahagian Pengurusan Sekolah Harian, Ministry of Education, Putrajaya, Malaysia; 4 Paediatric Department, Hospital Tunku Azizah Kuala Lumpur, Malaysia; 5 Port Dickson Health Clinic, Ministry of Health, Malaysia

Clinical and health guidelines are important documents that support health care providers, patients, and organisations by recommending models of care for daily practice. They are developed based on scientific evidence, yet their implementation (especially for guidelines on complex interventions) needs to take local context into consideration [1]. Specifically, these guidelines are typically based on evidence generated in developed countries, which have the substantial resources needed to conduct this research. This poses a significant challenge for the adaptation of evidence-based interventions and their effective implementation in low- and middle-income countries.

Qualitative studies offer insights into the processes through which programmes are implemented and can supply useful information about stakeholders’ experiences that can provide rich detail about policy contexts [2]. Our work exemplifies the impact of qualitative exploration in developing a Culturally Tailored school-based intervention for Asthma in Malaysia (CuT-AsthMa). This effort has influenced an initiative to develop and implement a national asthma guideline for schools in Malaysia.

## DEVELOPMENT OF CUT-ASTHMA

The World Health Organization recommends school-based asthma programmes to support asthma self-management among children in all settings [3]. Aligned with this recommendation, we used the UK Medical Research Council Framework for developing complex interventions to develop the CuT-AsthMa programme. The programme was based on systematic review evidence [4,5], local and international asthma guidelines, and findings from qualitative exploration among children with asthma, their parents, school staff, health care professionals, and policymakers [6–9].

This qualitative exploration was essential for adapting the CuT-AsthMa programme to the Malaysian context. Specifically, we conducted focus group discussions among children with asthma and their parents to understand how children manage their asthma at school. We found they were often hesitant to disclose they have asthma and that they had poor support to self-manage asthma at school [9]. Some children were reprimanded for bringing inhalers and spacers to schools because they were mistaken for toys. A few children were asked to use inhalers outside their classrooms because it was considered a distraction to others. Moreover, consultations with health care professionals often did not help children manage their asthma; children commonly described feeling disengaged (especially aged six to eight years) because information was directed to their parents rather than offering the child an opportunity to ask questions about looking after their asthma at school [9].

We then conducted interviews and focus group discussions with school staff, health care professionals, and policymakers to gather additional information for the CuT-AsthMa programme development [8]. We found that Malaysian schools do not have guidelines to support care for children with asthma. Moreover, school regulations limited children with asthma from participating in extracurricular and physical activities in school. Teachers had poor asthma awareness unless they had a personal history of asthma or family members with asthma. There were also misperceptions about asthma among the school staff and policymakers, with some believing, for example, that asthma symptoms and attacks can always be predicted and that asthma emergencies do not happen suddenly. Many teachers were uncertain about the action they should take if a child had asthma symptoms and an attack at school. A few teachers panicked when they faced these situations and would just call the parents to pick their children up from school.

The findings from the qualitative exploration delineated the core components of the CuT-AsthMa programme [10]. We designed it to include education and skills training aimed at improving children’s self-management; the school’s management of asthma emergencies; and general awareness within the school community. It focussed on providing important information about asthma and correcting common misperceptions, as well as training the school community to recognise asthma symptoms and act promptly and appropriately. We involved local experts; school teachers; and children with asthma and their parents to create the education materials. We tested the programme for feasibility, where the session on school’s management of asthma emergencies received good participation (88.7%) and feedback (86% rated good/excellent).

## DISSEMINATION OF FINDINGS TO STAKEHOLDERS

Few qualitative studies have been conducted with children in Malaysia. Our qualitative findings have demonstrated a compelling narrative on the significance of developing a school-based asthma programme in Malaysia. The voices of children in our study raised several important issues that needed attention and solutions. Considering this, we disseminated our findings to health care professionals, children with asthma, school staff, and policymakers.

In training courses with health care professionals, we emphasised the importance of communicating with the child during consultations on asthma. This included direct information gathering from children and counselling children and their parents on how to self-manage asthma at school. Furthermore, we engaged children with asthma through a children’s asthma club and co-designed an asthma activity book. During the book launch, we shared our findings with children, their parents, and the public to raise awareness about asthma and the importance of having asthma programmes in schools.

We highlighted the importance of continuing research in this area with other researchers and community engagement groups to gain more support for children with asthma. We presented our stakeholder engagement and dissemination efforts to the Malaysian Ministry of Education and Ministry of Health, who then endorsed the development of an asthma guideline for school children. These dissemination activities not only paved the way for programme implementation, but also established a network for future research and collaboration.

## DEVELOPMENT OF A NATIONAL SCHOOL ASTHMA GUIDELINE IN MALAYSIA

We held a two-day workshop in July 2023 to develop the national school asthma guideline. We invited key stakeholders from diverse backgrounds (school teachers, school health teams from government health clinics, health care professionals and policymakers from the Ministry of Health and Ministry of Education). In total, 38 people attended and group discussions were held in the workshop (**Figure 1**). School teachers shared their experiences and practical suggestions in the guideline design. School health teams and health care professionals provided their clinical expertise and proposed actions to support asthma care for children in school. Policymakers provided the group with relevant current circulars and regulations to ensure the guideline conformed with policies in both Ministries.

We divided the stakeholders into four groups to facilitate discussions. Each group comprised stakeholders from different backgrounds and was facilitated by a family medicine specialist with an interest in asthma or a paediatric respiratory physician. The topic for each group was based on findings of the qualitative exploration and recommendations of asthma guidelines in high-income countries, as follows: understanding asthma and recognition of children with asthma; actions when children have no asthma symptoms; actions when children have asthma symptoms; and actions when children have asthma attacks. After each group presented their key points, discussions were held with all stakeholders to reach a consensus. Implementation strategy was discussed and incorporated towards the end of the workshop.

After the meeting, we disseminated a draft of the guideline for stakeholders to review, which we then subsequently refined before sending it to external reviewers. We will send the final draft to the Ministry of Education for approval before disseminating it to the community (**Box 1**).

Box 1 **Learning points from the national guideline**

**Improve asthma awareness among school communities**

1. Provide asthma education, including symptoms, medication and address common misperceptions.

2. Identify children with asthma in schools and enhance student health records on asthma.

3. Incorporate the asthma guideline into current standard operating procedures of student health records.

**Encourage participation of children with asthma in school activities**

1. Encourage children with asthma to participate in regular school activities, including sports and physical activities with parental permission.

2. Allow children with asthma to participate in more vigorous physical activities, such as cross-country activities. Schools may request parents to obtain approval from the health care provider for these activities.

3. Enable children to take a dose of reliever inhaler prior to activities and ensure they carry it during the activities.

**Train teachers on appropriate action for asthma emergencies**

1. Support teachers by providing standard procedures for children who have asthma symptoms and attacks in school.

2. Train teachers to recognise different asthma presentations and administer reliever medication during asthma emergencies whilst calling for help.

**Implementation strategies**

1. Pilot the implementation of the guideline in some ‘early adopter’ schools.

2. Refine the programme based on feedback.

3. Roll out the programme nationally.

## CONCLUSIONS

Here we outlined how the processes of dissemination and stakeholder engagement using findings of qualitative studies led to the development of a national school asthma guideline in Malaysia. Qualitative explorations are essential for the adaptation of evidence-based interventions in low- and middle-income countries, offering a powerful narrative for social movement and informing policy decisions.
